# Nursing Management in Pediatric Intensive Care in South Asia

**DOI:** 10.3390/children12060726

**Published:** 2025-05-31

**Authors:** Daigo Hirao, Subrina Jesmin, Takehito Sugasawa, Adil Maqbool, Nobutake Shimojo

**Affiliations:** 1 Hirao Cardiovascular Clinic, Chiba 276-0042, Japan; jsubrina@gmail.com; 2 Faculty of Medicine, University of Tsukuba, Tsukuba 305-8577, Japan; 3 Health and Disease Research Center (HDRCRP), Bogra 5800, Bangladesh

**Keywords:** nursing management, hospital-acquired infections, pediatric intensive care units, liberation bundle, burnouts

## Abstract

Pediatric Intensive Care Units (PICUs) provide specialized care for critically ill children. Developing and managing these units in South Asia remains challenging. Resource limitations and infrastructural disparities are leading to challenging conditions. Above all, nurses play a pivotal role in delivering quality critical care. Effective nursing practices can curb hospital-acquired infections (HAIs), ensure medication safety, and enable protocols such as the ICU Liberation Bundle. In South Asia, another challenge is the proper management of the nursing workforce. Nurse-to-patient ratios are highly disproportionate, contributing to nurse burnout. This review highlights the country-specific challenges and circumstances. There is no one-size-fits-all solution; effective strategies vary based on each country’s context. With context-specific solutions, nurses can bridge the gap between healthcare teams and families, ultimately improving patient outcomes.

## 1. Introduction

The Pediatric Intensive Care Unit (PICU) is a relatively new subspecialty compared with adult intensive-care practice. It was first recognized in the USA during the 1980s, and the concept of a closed PICU system was established in 1993. A PICU is a dedicated ICU for infants and children in critical condition, requiring the latest medical equipment and a highly qualified multidisciplinary team to provide necessary care. Typically, a PICU manages major pediatric infections, severe injuries, organ failures, and other life-threatening emergencies. Early PICUs, such as the first unit in Sweden in 1955, extended knowledge from adult ICUs to treat children with pneumonia and sepsis [1]. For much of the twentieth century, admission criteria for PICUs remained broad (essentially any life-threatening illness requiring constant monitoring). Guidelines for setting up PICUs emphasized staff training, robust support services, and appropriate nursing-to-patient ratios as instrumental factors in delivering quality critical care. Over time, high-income countries achieved significant improvements in PICU care through technological advances and specialized workforce development [2].

Nurses are a key part of PICU patient care management. In pediatric ICUs, Healthcare-Associated Infections (HAIs) are among the most serious complications, strongly associated with increased morbidity. These infections originate within healthcare settings. Most patients arrive uninfected at admission. However, an HAI can appear within 48 h of admission after contact with contaminated individuals [3]. Contaminated medical devices are another common source [4]. Infections of this nature may lead to pressure ulcers, ventilator-associated pneumonia (VAP), and central line-associated bloodstream infections (CLABSIs). Historically, physicians were viewed as primarily responsible for preventing HAIs, but nurses can make sustainable improvements by implementing infection control interventions. For example, nurses’ vigilance in turning immobilized patients can reduce pressure ulcers, and their reminders to physicians about hand hygiene can lower HAI and mortality rates [5].

When comparing PICUs across regions, there are noticeable differences in the quality of care. North America has achieved dramatic advancements in technology and critical care over the past decades. Meanwhile, many low- and middle-income regions—including South Asia—continue to face limited resources; healthcare systems in these areas often struggle during outbreaks and pandemics. Now is the time to initiate a paradigm shift in South Asia’s PICU care to narrow the gap with global standards [6]. Similar challenges in developing pediatric critical care have been documented in other resource-limited regions, such as sub-Saharan Africa and Latin America, where common issues include workforce shortages and limited PICU beds [7]. However, Latin American PICUs have increasingly adopted family-centered practices—e.g., about 63% of units allow 24-h parental visitation, offering models that South Asian PICUs could learn from [8].

## 2. Methods

This article presents a narrative literature review of PICU nursing management in South Asia. We searched PubMed, Google Scholar, and English-language local journals for publications (2005–2025) addressing PICU organization, nursing workforce, patient safety, and outcomes in eight South Asian countries: Afghanistan, Bangladesh, Bhutan, India, Maldives, Nepal, Pakistan, and Sri Lanka. Key search terms included combinations of “pediatric ICU”, “intensive care nursing”, “South Asia”, “critical care outcomes”, and “nurse staffing”. We also reviewed relevant policy documents and World Health Organization reports. Articles and reports were included if they provided data or analysis on PICU structures, nursing roles, staffing ratios, training, or patient safety in these countries. To broaden the context, we compared findings with similar reviews from other regions (e.g., Africa, Latin America) when applicable. Because of marked heterogeneity in the available literature, we conducted a descriptive review and did not perform a formal quality assessment.

## 3. Overview of Pediatric Intensive Care in South Asia

Within Asia, this review focuses specifically on South Asia. The evolution of PICUs in these countries is only partially documented. India established its first PICU in the 1980s. Initially, the units operated from makeshift “treatment rooms.” There were limitations in the units; for instance, there was a lack of respiratory or hemodynamic support. Providing equipment for continuous monitoring was likewise difficult. Early Indian PICUs were poorly organized. It was in Chennai in 1991 that the first properly organized PICU was established. There were a total of seven beds located at the Kanchi Kamakoti Childs Trust Hospital [9].

**Figure 1:**
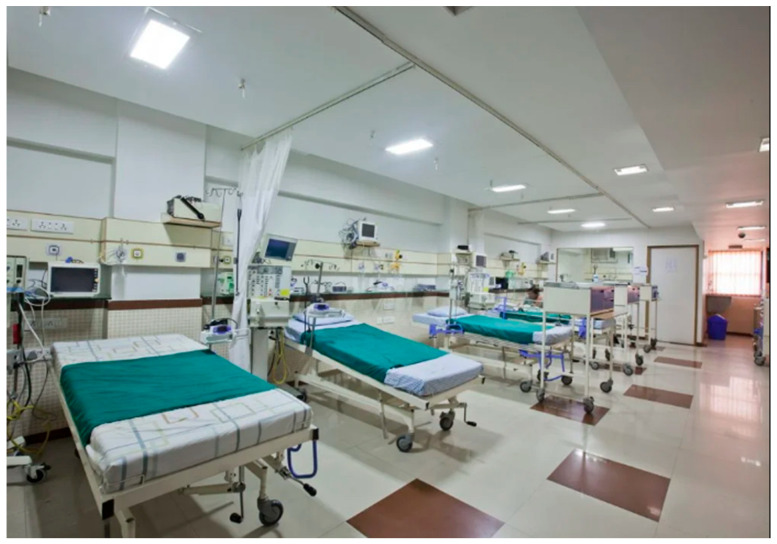
Demonstrates a typical Pediatric ICU in India; an image sourced with copyright permission from Children’s Hospital in Ahmedabad. A noticeable aspect here is the recruitment and training process of the entire team which had been led by one pediatric anesthesiologist.

Here, Figure 1 illustrates a typical Pediatric ICU in India. The source describes the unit’s staff-recruitment process in detail. The Apple Children’s Hospital PICU offers state-of-the-art care and support. It is the highest-ranked children’s hospital in Ahmedabad and the only one with a 22-bed PICU [10]. Since the emergence of the first PICU, the focus has been directed on expanding the unit capacity and training more doctors and nurses to deliver quality critical care. The teaching institutes have played a critical role in the process as they were responsible for establishing the formal PICU. In recent times, it has been reported that India is home to over 100 dedicated PICUs. The number includes both public and private facilities. Earlier units lacked proper support, and it remains challenging to maintain a recommended 1:1 nurse-to-patient ratio across all units. Training more pediatric critical care professionals remains crucial to sustain high-quality care [11].

In Bangladesh, the resources for critical care were initially far more limited than in India, and the PICU concept received little emphasis until the 1990s [12]. The country’s first ICU was set up in the 1980s at the National Institute of Cardiovascular Diseases [13], and the first PICU opened in 1994 at Dhaka Shishu (Children’s) Hospital [13]. As of the late 2010s, only around 100 hospitals in Bangladesh have any ICU facilities, ~80% of which are in the capital, Dhaka. Public hospitals account for just 27 ICUs (~22% of the total), while private ICU care is often prohibitively expensive for most of the population [14].

**Figure 2:**
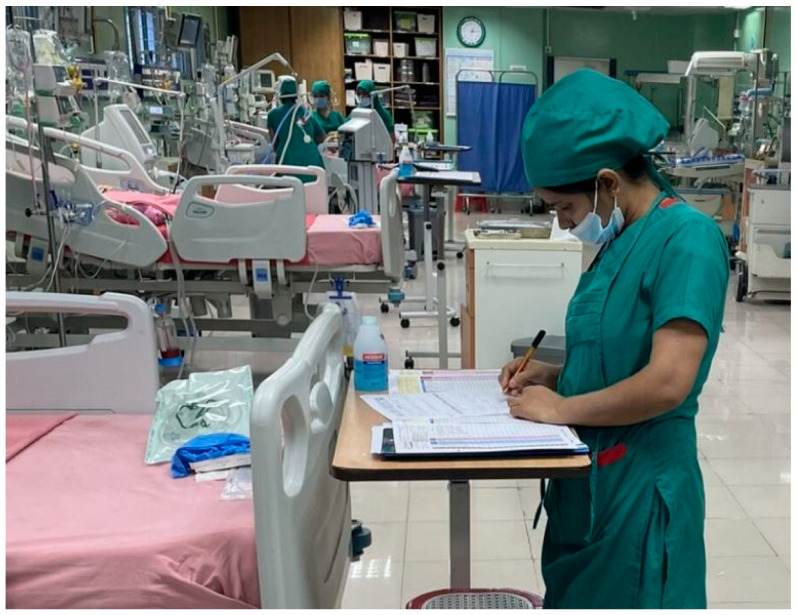
Presents a new PICU at the National Heart Foundation in Bangladesh, an image sourced with Copyright permission from Children’s HeartLink.org.

Bangladesh has only 11–12 hospitals with PICUs. The number is made up of both governmental and private hospitals [15]. Figure 2 highlights the continuous growth of critical care infrastructure in Bangladesh which is being aided by Children’s HeartLink at the National Heart Foundation in Mirpur, Dhaka, Bangladesh. It is imperative to note that Children’s HeartLink, a nonprofit organization has been working globally with PICU in different countries to train their respective doctors and nurses on pediatric heart care [16]. Only a few of these hospitals can reliably operate all PICU equipment. Bangladeshi PICUs also face severe nursing shortages (e.g., often 1 nurse per 4 patients), which compromise care quality. A small number of these hospitals are equipped with a dedicated team to manage the PICU. Establishing and managing PICUs in public hospitals remains a major government challenge. Private-sector PICUs are often unaffordable for many families. There is a lack of properly defined policies and protocols surrounding the management of pediatric intensive care units in Bangladesh [15].

Nepal’s first PICU opened in 1986 at Kanti Children’s Hospital, Kathmandu. During the early years, this PICU had just 4 beds [17]. There is a massive burden of critically ill children in the country. Consequently, appropriate facilities and trained specialists remain scarce.

**Figure 3:**
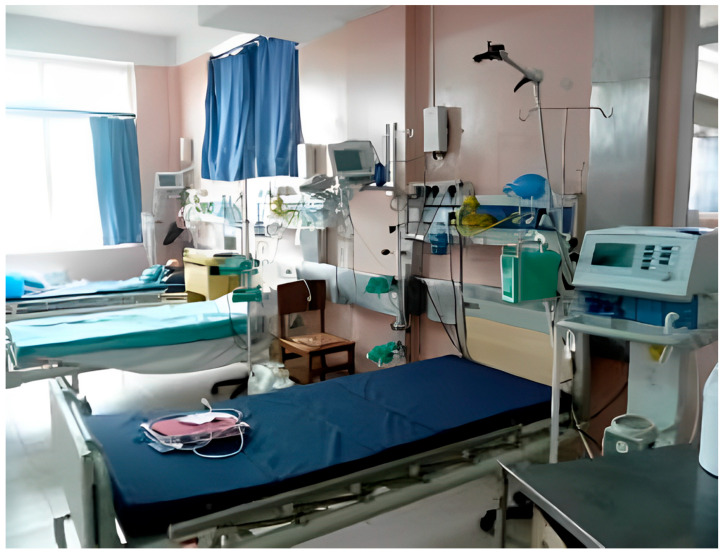
Shows an image of newly established PICU at Patan Hospital in Nepal, the image has been sourced with copyright permission sought from WFPICCS.com.

Currently, Nepal has 18 independent PICUs that admit nearly 2000 patients annually. Figure 3 underscores ongoing improvements in Nepal despite the lack of resources. However, a persistent skill gap and shortage of trained pediatric critical care staff continue to affect the quality of care. Nurse staffing in Nepal’s PICUs often exceeds recommended ratios, as limited personnel must care for a high volume of critically ill children, underscoring the need for specialized training programs [18].

The situation in Pakistan is slightly better than in Nepal and Bangladesh. A recent cross-sectional study assessed PICU capacity nationwide. Of 114 hospitals surveyed, 53 had a PICU. These units offered 667 PICU beds and 217 ventilators. Of the 53 hospitals, 38 are governmental hospitals and 15 are private. In 16 of these PICUs, there are a total of 20 intensivists with proper training. A 1:3 nurse-to-patient ratio—reported in 25 hospitals—is sub-optimal. Overall, it appears that a shortage of resources is hampering the quality of care in the PICUs in Pakistan. The lack of skilled nurses is also concerning for the PICU’s infrastructure in the country [19].

Based on the information available from the aforementioned countries (Table 1), a comparative table has been created to look at some of the differences in their infrastructures. The following Table is prepared in an attempt to better understand the situation in each of the aforementioned countries concerning the resources available to respective countries in terms of constructing and maintaining Pediatric Intensive Care Units (Table 1).

As shown above (Table 1; resourced from references [9,10,11,12,13,14,15,16,17,18,19]), a comparison of the Pediatric Intensive Care Units across South Asian countries highlights the disparities in infrastructure, staffing, and critical care capacity which are really alarming for the current global health issues. The information demonstrated in the Table 1 are extracted from the original sources. Here, it is important to note that, this type of healthcare information are often evident in the websites or messages from respective authorities or associations, report articles. Therefore, usual style of article referencing may not fit for the references used in this Table 1. We clearly state that Table 1 has been prepared by carefully reviewing the information related to the Pediatric ICUs [9,10,11,12,13,14,15,16,17,18,19] in the healthcare disadvantaged countries of South Asia. Due to insufficiency and unavailability of the information related to the PICUs in these South Asian countries, a great amount of challenges to assure the health services in PICU await the attention of global health authority. We believe context specific strategies should be designed from the local to global level for overcoming the challenges mentioned in the Table 1. In addition, the disparities and the differences seen in context of PICU in these countries deeply underscore the essence to tailor the strategies in each national context. 

## 4. Nursing Management in PICUs: Core Responsibilities

When children are admitted to a PICU, they often experience agitation, delirium, and sleep disruption. Children also suffer significant physical pain. For years, the Liberation Bundle has been used in ICUs for critically ill adult patients. The bundle comprises six elements: (1) pain assessment and prevention, (2) spontaneous awakening and breathing trials, (3) tailored analgesia and sedation, (4) delirium assessment and prevention, (5) early mobility and exercise, and (6) family engagement and empowerment.

These interventions improve outcomes in adult ICUs [20]. The Liberation Bundle is readily adaptable to PICUs. Nurses lead its implementation. In a limited resource setting, the nurses face quite a few hurdles. The care bundle, although underutilized, offers significant benefits if properly implemented. There are fewer complications when the bundle is implemented [21].

Nurses need to undergo regular professional development to handle complex cases in the PICU. When there are limited resources, it becomes difficult to receive staff training on such matters. This may result in inconsistencies in care practices [22]. There is also the issue of less research conducted on the application of the Liberation Bundle in PICUs. It only adds to the difficulty of not knowing the usefulness of the bundle for critically ill children [23].

The road to becoming a skilled and successful pediatric nurse to work in a PICU is filled with challenges. Nurses need to demonstrate a wide range of skills. There is a checklist related to the skills that nurses are required to have [24]. These are listed here:

As shown in Table 2, pediatric nurses need to demonstrate proficiency across a range of cardiovascular assessments and equipment handling skills.

As shown in Table 3, there are key criteria for endocrine disease assessment which have been outlined.

As shown in Table 4, there are detailed procedures and assessment tools for respiratory illnesses in pediatric patients.

As shown in Table 5, intravenous therapy skills are essential for PICU nurses, covering everything from peripheral IV insertion to the care of implanted vascular devices.

As shown in Table 6, there is an outline for assessing skills and procedures related to renal diseases that are frequently encountered during critical care.

As shown in Table 7, hematology and oncology nursing skills include chemotherapy administration, post-bone marrow care, and management of immunocompromised pediatric patients.

The nurses are required to show their proficiency in the above-mentioned criteria and more to be considered as proficient to work in a PICU. Simulation-based learning is a strategy that has been put to great use in studies involving pediatric nurses. In a particular study, future nurses achieved acceptable skills at post-tests for the nasogastric tube process. This achievement has been boosted by a simulation-based learning strategy. The study noted that the future nurses’ responses suggested that simulation-based learning could easily pave the way for creating well-rounded nurses [25].

Effective medication management, the critical component of pediatric intensive care, primarily falls into the responsibilities of the nurses. Errors with medication can pose a huge threat to the patient’s safety. The result may be serious harm, or if the case worsens, it may result in death. A study has been conducted recently on pediatric nurses and their knowledge of medication management. This study involved a total of 120 nurses. According to the study, a high number (70%) of the nurses are moderately aware of medication errors in children. There had been another study in the past that demonstrated that a significant number of nurses (61%) possessed average knowledge about medication errors. Both of the studies have been conducted in several states in India [26]. The study mentioned before it also raised questions regarding safe practices among nurses in PICUs. A large portion of the participants (80%) responded that they practiced sufficient safety measures. The rest reported that they were practicing with moderate safety. Medication management may be met with errors if the workload is high. In a limited resource setting, the patient-to-nurse ratio may be on the higher side. Moreover, the new nurses may show signs of unfamiliarity with the doctor’s handwriting. It may lead to medication errors. Given that resources are limited surrounding the critical care system, there is a need for medication administration guides. However, even that can be difficult to find at times [27].

When children become so ill that they must admitted to a PICU, they are automatically vulnerable to infection from the hospital. With the severity of the illness, it becomes difficult for children’s skin to provide regular physical defenses. Interruptions occur in the defense process as the children’s innate immunity along with adaptive immunity becomes compromised. Pediatric ICUs (PICUs) may encounter HAIs such as ventilator-associated pneumonia, bloodstream infections along catheter-associated urinary tract infections. Due to the onset of such infections, the patients are required to stay at the hospital for longer periods. It imposes severe health complications accompanied by a significant increase in care costs which creates further burden. The best way to mitigate the infection-related problem is to wash hands. Washing the hands properly leads to the prevention of nosocomial infections. While nurses have to be mindful about washing hands before and after dealing with critically ill children, the PICUs have a vital role to play. It is the responsibility of the unit itself to breed programs that require the workers to comply with hand hygiene. Additionally, PICUs in countries with abundant resources are slowly eliminating needless invasive devices. Pediatric nurses may need to undertake a multidisciplinary approach to minimize the risk of infection [28].

Meanwhile, another study has been conducted among pediatric nurses regarding infection control practices. The information was collected using a self-report questionnaire in a number of hospitals in Vietnam. A total of 102 nurses took part in the study, and the practices mentioned by them are: Use of protective devices, Disposal of sharps, Disposal of waste, Decontaminating spills along with used articles, and Taking preventive measures against cross-infection.

Infection control practices need to be considered vital to improve healthcare services. There needs to be some form of supervision that ensures that nurses always dispose of the sharps and wash their hands thoroughly [29]. With that said, pediatric nurses have several other responsibilities as well. One of those responsibilities is the emergency response and resuscitation of critically ill patients. Whenever patients go into cardiac arrest, nurses are the first professionals on the scene. Nurses are required to have excellent resuscitation skills. It is not always that simple to check the skill level of nurses for resuscitation skills. However, there is a “proficiency check” to diagnose the nurses’ skill level as being good enough to serve in a PICU. There is one study conducted on the nurses sharing their experiences regarding the proficiency check. The study revealed that those nurses had diverse experiences. Many nurses claimed to have a positive experience such that they had increased knowledge along with an increase in confidence. Meanwhile, some nurses had a negative experience associated with stress and anxiety [30].

## 5. Workforce Challenges and Solutions

Healthcare settings grow more complex every year. Any individual working in the healthcare sector is bound to face challenges. Nurses face both environmental and emotional challenges. The PICU is a workplace that can be stimulating and also rewarding at the same time. When nurses work in a PICU for a considerable amount of time, they become susceptible to compassion fatigue (CF) and at times secondary traumatic stress (STS). This situation often leads to burnout, reducing productivity and job satisfaction. CF has been described by experts as the cost that comes with caring. Secondary traumatic stress arises after prolonged exposure to patient suffering. The suffering is a result of a trauma or any life-threatening illness and is likely to result in burnout. In recent times, high-income countries have advanced in terms of medical technology. This has led to children living longer in the PICUs with chronic conditions. Working in this environment repeatedly exposes nurses to children’s suffering. They are also exposed to the traumatic experiences surrounding the death of those children. Additionally, they become exposed to emotional responses from their parents. When CF occurs, nurses become affected by physical health issues. Common consequences include depression, insomnia, fatigue, and anxiety. Compassion fatigue also lowers engagement and increases turnover [31]. 

With a high patient-to-nurse ratio, it is a given that the workload for nurses will increase. Nurses may monitor vital signs inconsistently. It may also lead to interventions being less effective. According to one study, high ratios reduce timely documentation of heart rate, oxygen saturation, and blood pressure. In addition, there is an association between a high patient-to-nurse ratio and the decreased ability to impact the level of care [32].

Nurses have to be allowed to voice their concerns in this setting. To find out more about the challenges that nurses face, a survey was carried out in the PICU of a hospital in Hong Kong. Other healthcare providers such as doctors and allied healthcare workers were invited to participate as well. There was moral distress among all three groups of professionals. According to a number of past studies, it is the nurses who display higher moral distress. Nurses experience greater moral distress because they enjoy less autonomy during ethical dilemmas. Nurses are often required to carry out care plans with which they disagree [33]. Studies report that up to 50 % of pediatric nurses perform tasks that conflict with their conscience. It says a lot about the power hierarchy in these units. The emotional burden is higher because nurses are the first in line to witness and experience the impact of a patient’s reaction to clinical decisions. There is more impact caused by the reactions of the patient’s families [34]. Since nurses are the first responders, they often have to bear the brunt of these reactions. The reactions may include insults and some form of hostile attitude from the family members if it is in Asian countries.

In the case of new PICUs, the aforementioned issues can lead to nurses perceiving lesser moral agency. There are other contributing factors as well, for instance, lack of proper team dynamics and lack of clarity about ethical climate. Most of these cases tend to occur in the newly established Pediatric ICUs [35]. The decrease in moral agency can directly result in moral distress among nurses. When moral distress occurs, it may leave nurses with no choice but to resign from their jobs. Studies into this issue are yet to fully confirm the direct relation between the two. There is always a possibility of other distressing factors burdening nurses with the decision to quit [36].

The experience of a nurse in care delivery in a PICU can help them navigate certain situations better. As mentioned above, nurses do experience a lack of autonomy over certain actions they have to perform. A working relationship exists between the nurses and the multidisciplinary team (MDT) inside the PICU. When nurses gain experience in the line of critical care, they are given the freedom to express their thoughts to the MDT. The MDT will grow to respect the nurses’ contribution as well [37]. On the other hand, if the nurses are not well informed about the patient’s primary information, it can cause misunderstandings with the patient’s family. Several studies involving pediatric nurses have found that there is a lack of appreciation for the nurses’ understanding of families. There are also actions that nurses take which may cause moral distress. Over time, there has been an increase in a nurse’s involvement in activities such as ventilation weaning, sedation managing as well, and feeding [38].

Burnout is rarely talked about in the case of pediatric nurses involved with critical care. There is however one study that dives into the issue of burnout among ICU nurses. According to the study, 1 out of 3 nurses working at an ICU undergo serious burnout. Burnouts are likely to lead to nurses taking “sick days” frequently or for prolonged periods. On the other hand, some nurses have reportedly fallen victim to depression and even substance abuse. It is never easy to address burnout in any industry. Burnouts are even more difficult to address in a healthcare setting [39].

Healthcare workers’ job satisfaction, including care provided by them directly associated with service satisfaction received from the patients’ side. The satisfaction amongst healthcare workers can be derived from the working environment and proper communication between the leader and the staff. It may also be derived from the management’s outlook and desire to solve problems. Oftentimes, the lack of these elements can lead to nurses becoming dissatisfied with their jobs. There is also a possibility that these issues can lead to burnout among the nurses. Anxiety can also result in job dissatisfaction and can lead to burnout [40]. Meanwhile, job dissatisfaction can play a key role in a person’s psychosocial functioning [41]. There is a negative impact of these two on the performance levels of pediatric nurses. According to one study, the increase in anxiety levels of healthcare workers, and nurses may result in the reduction of psychosocial functions. When pediatric nurses are involved, anxiety can prevent nurses from providing critical care in time. Several studies have gone on to discuss the emotional burdens carried by pediatric nurses. The mental health of a nurse is a vital aspect in ensuring critical care is provided to the patients. Nurses are not only affected by anxiety but also by depression, along with PTSD. If mental health is somehow in turmoil, it becomes difficult for pediatric nurses to handle workplace stress [42]. There is enough evidence from previous surveys to suggest that, the job dissatisfaction of nurses increases as they have to meet the extended needs of critically ill children along with their families [43]. It is a given that the workload of pediatric nurses has to be managed more efficiently to handle their mental well-being.

## 6. Cultural and Ethical Considerations in Nursing Care

In Iranian pediatric wards, nurses strive to understand parents’ cultural values. Studies describe how nurses work to comprehend and respect parents’ cultural needs. Today, culturally sensitive care is an implicit expectation in pediatric nursing. Nurses must deliver care that transcends ethnic and cultural boundaries. This mindset helps nurses reassure parents during their child’s critical PICU stay. Studies report that nurses consider cultural understanding and respect vital. These elements can build trust among the patient’s family [44]. Family-centered care helps forge strong nurse-family partnerships. Success with this model depends largely on nurses’ attitudes and experience. Therefore, understanding nurses’ perspectives is essential before implementation. This is especially true in developing countries, where nurses seldom have the chance to practise the model [45]. 

Palliative care often fails to reach all patients who need it. According to a report from the World Health Organization (WHO) in 2020, 25.7 million people needed palliative care and were at the end stages of their lives [46]. So, there has to be a more concentrated effort to ensure that more patients can receive it.

## 7. Technological and Policy Advancements

Pediatric healthcare is increasingly integrating Artificial Intelligence (AI) and telemedicine. These technologies are driving a rapid transformation in care delivery. They offer new opportunities to enhance both care quality and efficiency. AI-driven tools now help clinicians diagnose diseases early and guide timely interventions. Machine-learning and natural-language-processing algorithms analyze large datasets to detect disease patterns. Meanwhile, telemedicine has become more accessible for patients and families. Virtual consultations reduce the need for hospital visits. At the same time, patients can get access to doctors when necessary without having to travel from remote areas as in the past. Meanwhile, AI can also access the real-time data from the aforementioned consultations to refine the care plan. Perhaps the finest example of the partnership between AI and telemedicine is India’s eSanjeevani platform. To date, eSanjeevani has facilitated care for more than 241 million patients across several countries. With the platform, it is now possible to reach patients who live in remote areas [47]. However, the influx of technology brings its challenges. One major concern is alarm fatigue: nurses are bombarded with frequent monitor alarms and ventilator alerts, which can desensitize staff over time. This desensitization may lead to slower responses to true emergencies, posing a patient safety risk [32]. Addressing alarm management through smarter devices or alarm reduction protocols is therefore an important aspect of technological advancement in PICUs.

While the level of critical care in India is improving, the Low and Middle-Income Countries (LMICs) are struggling in that same aspect. In many LMICs, participation in critical-care training remains minimal. Much of the responsibility then falls on the shoulders of critical care societies, along with education networks, to create curricula about critical care. Evidence-based procedures are seldom practiced. For these countries, it is now crucial to rely on critical care societies, if they exist, to develop short courses on critical care. Locally appropriate critical-care models should be developed through targeted research. This is where governments can extend their support through financial means and policy development to build up critical care. Governments have an integral role to play when it comes to infrastructural support. Once infrastructure is in place, the critical-care community must collaborate with health authorities to realize its full potential [48]. Understanding nurses’ perspectives on pediatric intensive care is equally important. A 2022 survey queried 146 PICU nurses from eight Asian countries. As part of the study, they were provided with seven research domains and asked to provide research topics. After some time, the nurses narrowed down the research domains into three: end-of-life care, professionalism, and lastly managing pain, sedation along delirium. These are the key themes according to these nurses to conduct further research on. Moreover, the themes have to be taken into consideration for conducting further training sessions for nurses [49].

## 8. Implications for Practice and Policy

Working in a PICU often leads to high nurse turnover. Each day, nurses manage complex situations; for example, critically ill children can undergo decompensation, a sudden clinical decline. During decompensation, children become hemodynamically unstable. Nurses must respond rapidly with resuscitation to save the patient [50]. As first responders in such crises, they must draw on all their training. Managing these situations imposes a considerable burden. Given the constant pressure in a Pediatric ICU, measures are needed to keep turnover low and support high-quality care. Units could create well-being noticeboards and schedule psychological drop-in sessions. They can also adopt the Nurse Wellbeing Index (NWI) for nurse self-assessment. NWI scores enable continuous monitoring of nurse well-being. If scores are unsatisfactory, targeted interventions are required to prevent turnover [51].

Several actions and policies can enhance PICU nursing management in South Asia. First, units can implement Liberation Bundle protocols. This approach should include early mobilization programmes and integrated physiotherapy. Family-centred care models should also be adopted because they reduce delirium rates. Continuous professional development for nurses is equally important. PICUs can offer medication-safety workshops focusing on high-alert drugs and dose-calculation checks [52]. Maintaining a 1:1 nurse-to-patient ratio for high-acuity cases is essential. Finally, PICUs could introduce automated hand-hygiene monitoring systems. These systems aim to reduce infections, to which critically ill children are especially vulnerable.

## 9. Future Research Directions

Table 8 was compiled after a careful review of the studies cited within it. Its entries reflect each country’s context and the specific challenges encountered. The table also serves as a roadmap, highlighting topics that warrant deeper investigation.

Table 8 outlines four high-priority domains for future pediatric ICU research in South Asia and lists an illustrative key question for each. The Workforce Outcome domain emphasizes rigorous, multi-center studies that quantify how varying nurse-to-patient ratios influence mortality and other clinical outcomes. Infection Control calls for implementation-science projects that test sustainable, low-cost interventions—such as automated hand-hygiene monitoring or nurse-led device-care bundles—to reduce VAP and CLABSI rates. The Well-being of Nurses domain highlights the need for longitudinal cohort studies that track burnout trajectories over time, identifying modifiable workplace factors that affect retention and psychological health. Finally, the Liberation Bundle Implementation domain seeks pragmatic trials evaluating the feasibility and clinical impact of adapting the ICU Liberation (ABCDEF) Bundle to low- and middle-income PICU settings, with particular attention to resource constraints and cultural considerations. Collectively, these four research tracks provide a roadmap for evidence generation that can inform policy, optimize patient safety, and strengthen the PICU nursing workforce across South Asia.

## 10. Discussion

The act of managing PICUs in South Asia with the help of highly skilled nurses is not an easy feat. There are challenges involved that make the management of it all so time-consuming. Another table (Table 9) has been prepared in light of the existing issues observed from previous studies well referenced in this paper.

The table above (Table 9) outlines the existing issues in South Asian PICUs. These issues range from resource shortages and staffing imbalances to hospital-acquired infections. There are also inconsistencies in the use of protocols. While challenges persist, opportunities remain to enhance PICU performance and nurses’ working conditions. Administrators should invest in professional development programs for PICU nurses. These programs help identify skill gaps and promote adherence to evidence-based practices. Crucially, training should focus on building specialized skills needed in PICUs—such as pediatric resuscitation, ventilator management, hemodynamic monitoring, and effective communication with families. Simulation-based training boosts nurses’ resuscitation skills and confidence; incorporating it routinely can better prepare staff for critical events. Nursing schools and education networks in South Asia should integrate pediatric critical-care content into their curricula, ensuring new nurses enter the workforce with fundamental PICU competencies. Continuous in-service training and simulation exercises can reinforce these skills and keep staff up-to-date with best practices. Collaboration with international PICU networks may help South Asian units share knowledge and improve standards. Governments have an integral role as well—by providing financial support and clear policies, they can facilitate the development of critical-care infrastructure and standardized protocols across hospitals. For instance, mandating minimum nurse-staffing levels and earmarking funds for training would tackle systemic gaps. Strong nurse-to-patient ratio policies (with 1:1 care for high-acuity patients as a goal) and medication-safety protocols (such as double-checking pediatric drug dosages) must be instituted. Multidisciplinary teamwork should be encouraged; when physicians, nurses, and other professionals work in unison, patient outcomes markedly improve.

## 11. Conclusions

Nursing management in South Asian PICUs faces numerous challenges, including resource limitations, uneven infrastructure, and workforce skill gaps. To improve critical-care quality, policymakers must adopt and enforce evidence-based practices—for example, rolling out the PICU Liberation Bundle nationwide, setting infection-control standards, and investing in workforce development. At the practice level, PICUs should enhance their nursing workforce through ongoing training and professional development; ensuring nurses are prepared for any situation. Robust infection-control measures—aimed at reducing VAP, CLABSI, and other HAIs—together with strong medication-safety initiatives, must be implemented with clear backing from hospital leadership. It is also imperative to address nurse staffing: concerted action is required to achieve a safe 1:1 nurse-to-patient ratio for the most critical cases and to foster family-centered care models (including enabling parental presence when possible). These steps will not only improve patient care but also help prevent nurse burnout.

These findings clarify the implications for policy, practice, and research. Governments and health ministries should develop clear guidelines and accreditation standards for PICUs—including nurse staffing ratios, training requirements, and safety audits—and allocate funding to strengthen pediatric critical-care capacity. PICU managers should introduce family-friendly policies and comprehensive support systems for nurses—such as counseling services and wellness programs—to boost retention and job satisfaction, thereby enhancing patient safety. Regular simulations and refresher training can sustain high clinical performance. Future studies should prioritize evaluating interventions in limited-resource settings via multicenter trials. Longitudinal research on nurse burnout and staffing, as well as mixed-methods studies on PICU workforce challenges, is needed to inform locally relevant solutions. Pursuing these aligned policy, practice, and research agendas will steer South Asian PICUs toward sustainable advances in care delivery. In the end, better-supported nurses and evidence-based management will translate into improved patient outcomes and a stronger pediatric critical-care system.

## Figures and Tables

**Table 1:** Pediatric Intensive Care Units in South Asia.

Country	First PICU	Current PICU Availability	Key Institutions & Statistics	Challenges
India [9,10,11]	1991 (Chennai, KKCTH) [9]	>100 PICUs (public + private) [11]	7-bed PICU at KKCT Hospital (first organized unit); led by a pediatric anesthesiologist; teaching institutes crucial [9]	Earlier units lacked support; maintaining a 1:1 nurse-patient ratio remains challenging; training more professionals is crucial
Bangladesh [12,13,14,15,16]	1994 (Dhaka Shishu Hospital) [12]	11–12 PICUs (public + private) [15]	First ICU in the 1980s at NICVD; 27 government ICUs (22%); 80% of ICUs in Dhaka. [14]	Urban-rural disparity; lack of protocols; private PICUs unaffordable; nurse staffing is inadequate (often ~1:4 nurse: patient)
Nepal [17,18]	1986 (Kanti Children’s Hospital) [17]	18 independent PICUs [18]	Started with 4 beds; ~2000 annual PICU patients; high critical care burden [18]	Shortage of trained staff; skill gaps in care; staffing often falls short of recommended levels
Pakistan [19]	N/A (first survey 2023) [19]	53 PICUs (of 114 hospitals) [19]	667 beds, 217 ventilators (nationwide); 38 govt + 15 private PICUs; 16 units with 20 trained intensivists; nurse:patient ~1:3 [19]	Resource shortages; lack of skilled nurses limits infrastructure development

**Table 2:** Cardiovascular Disease Assessment Criteria for Pediatric Nurses.

Criteria	Assessment	Procedures/Equipment	Access and Line
Cardiovascular	Head-to-Toe	Cardiac Monitor	Care of Central Venous Catheter
	Heart Sounds	CPR of Infant	Care of Subclavian Lines
	Pulses	CPR of Child	Care of Arterial Catheter
	Wolf-Parkinson White Syndrome	Preparation of Emergency Drugs	ECMO therapy
	Perfusion	Defibrillation	Autotransfusion system
	Acyanotic and Cyanotic Heart Disease	Rhythmic recognition	Care of Swanz Ganz Catheter

**Table 3:** Endocrine Disease Assessment for Pediatric Nurses.

Criteria	Assessment/Equipment/Skills
Endocrine Disease	Diabetes/Ketoacidosis
	Glucose Monitoring Devices
	Insulin Drip

**Table 4:** Respiratory Disease Assessment and Equipment Assessment Criteria for Pediatric Nurses.

Criteria	Assessment and Treatment	Procedures and Equipment	Care of Patient on Oxygen Treatment
Respiratory Disease	Asthma	Intubation/Extubation	Mask/Cannula
	Cystic Fibrosis	Tracheotomy Care	Ambu Bag
	Pneumonia	Capillary Blood Gas	Ventilator
	Epiglotitis	Arterial Blood Gas	Apnea Monitor
	Chest Tubes	Interpretation of Blood Gas	Endo/Naso Tracheal Tube
	N/A		O_2_ Analyzer
			Pulse Oximeter
			Nasal tracheal suctioning
			Endo tracheal suctioning

**Table 5:** Intravenous (IV) Therapy-Related Experience and Skill Criteria for Pediatric Nurses.

Criteria	Experience and Skill
Intravenous (IV) Therapy	Peripheral IV Insertion (Angio/Intracath)
	Administration of Blood/Blood Products
	Care of Implanted Vascular Access
	Insertion of Scalp Veins
	PICC Lines

**Table 6:** Renal Disease Assessment Criteria for Pediatric Nurses.

Criteria	Assessment/Equipment/Skills
Renal Disease	Foley Catheter Disease
	Hemodialysis
	Renal Transplant
	Peritoneal Dialysis

**Table 7:** Hematology/Oncology Disease Assessment Criteria for Pediatric Nurses.

Criteria	Assessment/Treatment Disease
Hematology/Oncology Disease	Anemia
	Hemophilia
	Administration of Chemotherapy
	Immunocompromised patients
	Post Bone Marrow Treatments

**Table 8:** Future Research Directions.

Research Focus Area	Key Questions
Workforce Outcome	Impact of nurse-patient ratio on mortality [11,19]
Infection Control	Minimizing infection rate by sustainable means [5,29]
Wellbeing of Nurses	Longitudinal burnout trajectories [31,39,40]
Liberation Bundle Implementation	Effectiveness of Liberation Bundle in LMICs in South Asia [21,23,52]

**Table 9:** Existing Issues of PICUs in South Asia.

Issue	Scenario in Multiple Countries
Limited resources & infrastructure	Disparities exist across South Asia. For instance, India has ~100 fully functional PICUs, whereas Bangladesh struggles with only 11–12 although the exact number of PICUs cannot be stated here concretely due to ethical issues, privacy, and confidentiality of the information originating from the various tiers from governmental to private hospitals. Nepal and Pakistan similarly lack sufficient units and skilled personnel.
Nurse–patient ratio & burnout	The ratio is about 1:3 in many Pakistani PICUs, making it difficult for nurses to simultaneously care for multiple critically ill children, leading to burnout and high turnover. Similar staffing shortfalls are seen across the region (e.g., ~1:4 in Bangladeshi PICUs), exacerbating nurse fatigue and compromising patient care.
Hospital-acquired infections (HAIs)	PICUs encounter frequent HAIs (e.g., ventilator-associated pneumonia and central line-associated bloodstream infections (CLABSI)). Nurses must rigorously implement infection control to keep infection rates in check given patients’ critical conditions.
Lack of evidence-based protocols	Evidence-based protocols like the Liberation Bundle are rarely implemented in South Asian PICUs, reflecting gaps in translating knowledge into practice.
Limited family involvement	Parental presence in PICUs is often restricted due to space or policy constraints. By contrast, ~63% of Latin American PICUs permit 24-h parent visitation. Limited family engagement can heighten children’s anxiety, whereas involving parents in care (when feasible) helps reduce stress and improve coping.
Patient safety and quality gaps	Many units lack robust patient safety monitoring. Medication errors and ICU infections (VAP, CLABSI) are prevalent concerns, yet safety indicators are not systematically tracked. Inconsistent infection control compliance and limited medication safety training for nurses highlight the need for stronger quality assurance programs.

## Data Availability

Not Applicable, well stated through the manuscript.
